# Comparative study on the cytotoxic effects of benzalkonium chloride on the Wong-Kilbourne derivative of Chang conjunctival and IOBA-NHC cell lines

**Published:** 2008-03-04

**Authors:** E. Brasnu, F. Brignole-Baudouin, L. Riancho, J.-M. Warnet, C. Baudouin

**Affiliations:** 1 Department of Ophthalmology III, Quinze-Vingts National Ophthalmology Hospital, Paris, France; 2 Department of Toxicology, Faculty of Biological and Pharmacological Sciences, Paris Descartes University, Paris, France; 3 INSERM UMR S 872, Cordeliers Biomedical Institute, Paris Descartes University, Paris, France

## Abstract

**Purpose:**

The Wong-Kilbourne derivative of Chang conjunctiva-derived cell line has been widely used for toxicological and functional in vitro studies on the ocular surface. The common reserve to this cell line is the reported contamination with HeLa cells. Thus, the IOBA-NHC spontaneously immortalized conjunctival epithelial cell line has been recently developed and did not show other cell type contamination. Our purpose was to determine whether both cell lines would be equally suitable for in vitro toxicological studies. Therefore, we compared in these two cell types the toxic effects of the preservative, benzalkonium chloride (BAC); its toxicity has been often reported on conjunctival in vivo and in vitro models.

**Methods:**

The necrotic, apoptotic, and oxidative effects of BAC were evaluated on Chang and IOBA-NHC cell lines using microplate cytofluorometry tests (neutral red, 2,7- dichlorofluorescein diacetate dye [H_2_DCF-DA], hydroethidine, and Yopro-1), flow cytometry (Annexin V/7-AAD and DNA content tests), and standard immunofluorescence stainings. Cells were exposed to five concentrations of BAC (10^−2^%, 5.10^−3^%, 10^−3^%, 10^−4^%, and 10^−5^%) for two incubation times: 15 min of treatment and 15 min of treatment followed by 24 h of cell recovery in complete medium.

**Results:**

All parameters of toxicity increased in a BAC dose-dependent manner on both cell lines.

**Conclusions:**

The comparison of BAC toxicity on both cell lines supported the use of IOBA-NHC and Chang cells for toxicological in vitro studies. Drawbacks of both cell lines have to be known and considered in studies performed on these cell lines.

## Introduction

One of the best ways to investigate the ocular surface, especially the conjunctival epithelium, is theoretically to get human conjunctival biopsies that provide epithelial, stromal, inflammatory, and goblet cells ex vivo [1]. However, these samples are traumatic and cannot be used for research purposes in benign conditions. Less invasive techniques like conjunctival impression cytology or brush cytology have been developed that allows immunological, physiopathological, and toxicological studies but collect a limited number of cells [2-5]. Therefore, cell cultures have been widely used for investigating some pathophysiological aspects of the human conjunctiva. Particularly, cell lines permit easy and quick cell development and have the advantage over primary culture of being independent from conjunctival biopsy availability. Currently, the Wong-Kilbourne derivative of Chang conjunctival cell line has been widely used for toxicological and functional in vitro studies on ocular surface diseases. This cell line has been immortalized from normal human conjunctival epithelial cells but presents some characteristics distinguishing it from normal tissue and primary cultures, particularly in their response to inflammatory cytokines like tumor necrosis factor (TNFα) and interferon (IFNγ) [6]. Indeed, like other cell lines, it has acquired some differences with normal conjunctiva in its phenotypic characteristics. But the most common reserve to this cell line is the reported contamination with HeLa cells [7]. Recently, Diebold et al. [8] characterized the IOBA-NHC spontaneously immortalized cell line that showed no other cell type contamination but had some phenotypic differences with the normal conjunctival epithelium. Our purpose was to evaluate the relevance of the IOBA-NHC cell line in toxicological research studies, determining whether these cell lines would be fully comparable and suitable for toxicological in vitro studies. As benzalkonium chloride (BAC) is a preservative widely used in ophthalmic medications and is known to induce toxic effects on conjunctival cells in vitro and in vivo [9-12], its necrotic, apoptotic, and oxidative effects were evaluated and compared in the present study on both cell lines.

## Methods

### Conjunctival cell lines

IOBA-NHC cells were cultured under standard conditions (humidified atmosphere of 5% CO_2_ at 37 °C) in DMEM/F12 supplemented with 1 µg/ml bovine pancreas insulin, 2 ng/ml mouse epidermal growth factor, 0.1 µg/ml cholera toxin, 5 µg/ml hydrocortisone, 10% fetal bovine serum (FBS), 50 UI/ml penicillin, and 50 UI/ml streptomycin, as previously described [8].

The Wong-Kilbourne derivative of Chang conjunctival cells (clone 1 to 5c-4l American Type Culture Collection [ATCC, Manassas, VA]-certified cell line [CCL], 20.2) were cultured under standard conditions (humidified atmosphere of 5% CO_2_ at 37 °C) in Dulbecco’s minimum essential medium supplemented with 10% fetal bovine serum, 1% glutamine, 50 UI/ml penicillin, and 50 UI/ml streptomycin.

Cells from passages 5–50 were used in all experiments. Every day, normal culture development was controlled by phase-contrast microscopy. Cells were removed by gentle trypsin incubation at confluence then counted. Then, they were seeded into 96 well culture plates (Corning, Schiphol-Rijk, the Netherlands) for microtitration analysis (5,000 cells per well), in 12 well culture plates (TPP, Trasadingen, Switzerland) for flow cytometric analysis (25,000 cells per well), and on slides (Lab-tek II chambered coverglass; Nunc International, Napierville, IL) for standard immunofluorescence experiments. Cultures were kept at 37 °C for 24 h. Subconfluent cells (culture surface covering nearly 70%) were then exposed to the different BAC concentrations.

### Cell treatments

Benzalkonium chloride was dissolved in phosphate buffered saline (PBS). Five different concentrations of BAC (10^−2^%, 5.10^−3^%, 10^−3^%, 10^−4^%, and 10^−5^%) were analyzed. Indeed, these concentrations are equivalent to or lower than the concentrations of most available eye-drops. Concentrations of 10^−1^% and higher were not tested as they are known to be excessively toxic on conjunctival cells in vitro, inducing cell lysis immediately after treatment [10]. According to previously validated studies under the same protocol [11], two incubation times were applied to the cells: 15 min of treatment and 15 min of treatment followed by 24 h of cell recovery in complete medium. The 24-h cell recovery period was tested to approach the clinical conditions in which the conjunctival tissue may recover after eye drop instillation.

### Microplate cytofluorometry

Microplate cytofluorometry was performed on the Saphire Microplate reader (Tecan Instruments, Lyon, France). Four different assays were used according to previously validated methods [11,13-21]. As previously described, neutral red stain (Fluka, Ronkonkoma, NY) was used to evaluate membrane integrity, closely correlated with cellular viability [15]. Reactive oxygen species (ROS), particularly H_2_O_2_, were detected using the 2,7- dichlorofluorescein diacetate dye (H_2_DCF-DA; Molecular Probes, Eugene, OR) [16], and the hydroethidine probe was used to detect the superoxide anion (O_2_^.-^) production (Molecular Probes, Eugene, OR) [17]. The Yopro-1 probe (Molecular Probes, Eugene, OR) was used to evaluate apoptosis as previously described, showing the opening of specific membrane pores through the P_2_X_7_ cell death receptor activation [18-21]. In all experiments, sterile phosphate buffered saline (PBS) was used as the control. Microplate cytofluorometry results were obtained in fluorescence units and were expressed as a percentage of the control. Each drug was tested in six wells (50 µl per well), and each experiment was performed in triplicate. Hydroethidine, H_2_DCF-DA, and Yopro-1 results were expressed using a ratio of the results of these tests to those of the neutral red test to correlate them to cellular viability.

### Flow cytometry

Annexin/V-7AAD staining and DNA content analysis are two apoptotic tests that were assessed by flow cytometry on a FC 500-CXP flow cytometer (Beckman Coulter, Miami, FL) equipped with an argon laser emitting at 488 nm [22,23]. Annexin V binds specifically to phosphatidylserines that shift to the outer leaflet of the plasma membrane during the early apoptotic process. 7-amino-actinomycin D (7-AAD) is a fluorescent probe that intercalates into nucleic acids of late apoptotic or necrotic cells. After 24 h of culture in 12 well plates at 37 °C, cells were incubated with the different BAC concentrations for 15 min. Then, they were incubated with ethylenediamine tetraacetic acid (EDTA) at 1 mM, collected, and suspended in 1 ml PBS. Annexin V and 7-AAD were used as recommended by the manufacturer (Beckman Coulter, Immunotech, Luminy, France). A biparametric histogram was performed to discriminate annexin V and 7-AAD, which would determine four cell populations: cells negative to both markers (normal viable cells), cells positive only to annexin V (early apoptotic), cells positive to both annexin V and 7-AAD (late apoptotic), and cells positive only to 7-AAD (necrotic).

Alteration of DNA content was measured through the sub-G_1_ peak flow cytometric analysis on a FL4-fluorescence histogram in a linear mode to characterize the late apoptosis process. After 15 min of BAC treatment followed by 24 h of cell recovery in complete medium, cells were collected using EDTA as previously described and fixed with 0.5% paraformaldehyde (PFA) fixative in PBS at 4 °C for 24 h. After the 24 h fixation, samples were washed with cold PBS, permeabilized in 0.1% saponin, stained with 50 µg/ml propidium iodide (Interchim, Montluçon, France), and analyzed on the flow cytometer. The sub-G_1_ region was determined by a cursor defined in the controls and excluding the debris as described previously [23].

### Immunocytology

Standard immunofluorescence staining under a confocal epifluorescence microscope (E800, PCM 2000; Nikon, Tokyo, Japan) was performed to assess morphologic patterns of cells. Cells were cultured on chamber slides (Lab-tek; Nalge Nunc International, Rochester, NY) and incubated with the different BAC concentrations for 15 min. They were then washed with PBS and fixed for 15 min with 4% PFA in PBS at 20 °C. Fluorescent phalloidin (200 U/ml; Alexa 488, Molecular Probes) was then added to detect F-actin. After 30 min of incubation with phalloidin, cells were washed in PBS before propidium iodide staining and microscopy examination.

### Statistical analysis

Statistical comparisons were performed with Sigma Stat 2.0 (SPSS, Chicago, IL). For the microplate cytofluorometry data, significance was assessed using one-way analysis of variance (ANOVA) followed by the Dunnett test. The flow cytometry data were analyzed using one-way ANOVA followed by the Bonferroni *t*-test.

## Results

### Cell viability (neutral red probe)

**Figure 1:**
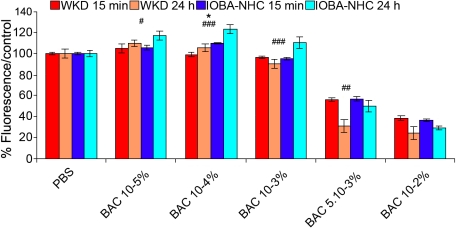
Membrane integrity evaluation using the neutral red test on Chang (WKD) and IOBA-NHC cells. This figure shows the membrane integrity evaluation using the neutral red test (microplate cytofluorometry) on Chang (WKD) and IOBA-NHC cells after two incubation times, 15 min of treatment with different BAC concentrations (WKD 15 min, IOBA-NHC 15 min) and 15 min of treatment with different BAC concentrations followed by 24 h of cell recovery in complete medium (WKD 24 h, IOBA-NHC 24 h). Note that membrane integrity decreased in a concentration-dependent manner on both cell lines. The asterisk denotes statistically significant differences between the two cell lines after 15 min of treatment (p<0.01), and the sharps (hash marks) denote statistically significant differences between the two cell lines after 24 h of cell recovery (### p<0.001, ## p<0.01, # p<0.05). BAC, benzalkonium chloride; PBS, phosphate buffered saline. Means±SEM.

After a 15 min incubation time, the membrane integrity decreased in a concentration-dependent manner on the two cell lines (Figure 1). Thus, on the Chang cell line, the most important toxicity was obtained with BAC 10^−2^% (38% of the control, p<0.001 versus control) followed by BAC 5.10^−3^% (56%, p<0.001) then BAC 10^−3^%, 10^−4^%, and 10^−5^% (95%, 98%, and 104%, respectively, p=NS [not significant]). On IOBA-NHC cells, membrane integrity results were 36% of the control with BAC 10^−2^% (p<0.001), 56% with BAC 5.10^−3^% (p<0.001), 95% with BAC 10^−3^% (NS), 109% with BAC 10^−4^% (p<0.001), and 106% with BAC 10^−5^% (p<0.05).

After 24 h of cell recovery (Figure 1), cellular viability increased on both cell lines with BAC at 10^−4^% and 10^−5^%. With BAC 10^−3^%, cellular viability increased on IOBA-NHC cells and decreased on Chang cells while with BAC 5.10^−3^% and 10^−2^%, cellular viability decreased on both cell lines.

### Reactive oxygen species detection (H_2_DCF-DA probe)

**Figure 2:**
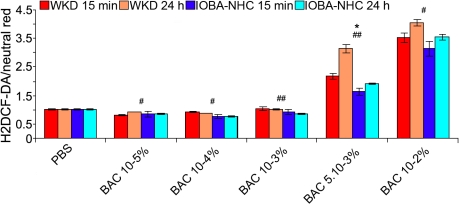
Reactive oxygen species detection using the H_2_DCF-DA test on Chang (WKD) and IOBA-NHC cells. This figure shows the reactive oxygen species (ROS) detection using the H_2_DCF-DA test (microplate cytofluorometry) on Chang (WKD) and IOBA-NHC cells after two incubation times, 15 min of treatment with different BAC concentrations (WKD 15 min, IOBA-NHC 15 min) and 15 min of treatment with different BAC concentrations followed by 24 h of cell recovery in complete medium (WKD 24 h, IOBA-NHC 24 h). Note that the highest ROS production was obtained with the highest BAC concentrations and varied in a concentration-dependent manner on both cell lines. The asterisk symbol denotes statistically significant differences between the two cell lines after 15 min of treatment (*p<0.01), and the sharps (hash mark) denote statistically significant differences between the two cell lines after 24 h of cell recovery (##p<0.001, #p<0.01). BAC, benzalkonium chloride; PBS, phosphate buffered saline. Means ± SEM.

After a 15-min exposure time, the highest ROS production was obtained with the highest BAC concentrations and varied in a concentration-dependent manner (Figure 2). Indeed, on Chang cells, the highest ratio was obtained with BAC 10^−2^%, (3.52, p<0.001 versus control) followed by BAC 5.10^−3^% (2.17, p<0.001) then BAC 10^−3^%, 10^−4^%, and 10^−5^% (1.05 [p=NS], 0.91 [p=NS], and 0.8 [p<0.001], respectively). On IOBA-NHC cells, the highest ratio was also obtained with BAC 10^−2^% (3.11, p<0.001) followed by BAC 5.10^−3^% (1.62, p<0.001), BAC 10^−3^% (0.91, p=NS), 10^−4^% (0.76, p<0.01), and 10^−5^% (0.84, p=NS).

After 24 h of cell recovery (Figure 2) in both cell lines, small changes of ROS production were observed with BAC concentrations of 10^−3^% and lower whereas ROS production increased with BAC 5.10^−3^% and BAC 10^−2^%.

### Superoxide anion detection (hydroethidine probe)

**Figure 3:**
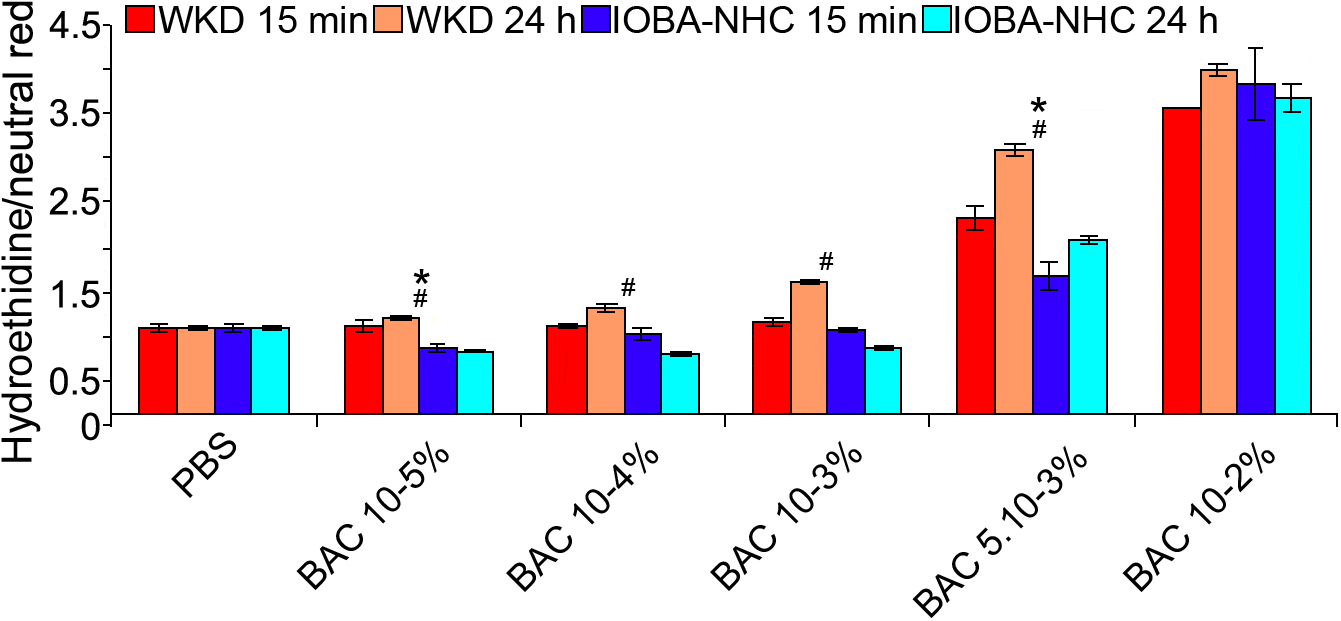
Superoxide anion detection using the hydroethidine test on Chang (WKD) and IOBA-NHC cells. This figure shows the superoxide anion detection using the hydroethidine test (microplate cytofluorometry) on Chang (WKD) and IOBA-NHC cells after two incubation times, 15 min of treatment with different BAC concentrations (WKD 15 min, IOBA-NHC 15 min) and 15 min of treatment with different BAC concentrations followed by 24 h of cell recovery in complete medium (WKD 24 h, IOBA-NHC 24 h). Note that the highest superoxide anion production was obtained with the highest BAC concentrations and varied in a concentration-dependent manner on both cell lines. The asterisk symbol denotes statistically significant differences between the two cell lines after 15 min of treatment (*p<0.05), and the sharp (hash mark) denotes statistically significant differences between the two cell lines after 24 h of cell recovery (#p<0.001). BAC, benzalkonium chloride; PBS, phosphate buffered saline. Means ± SEM.

After a 15 min exposure time, the highest superoxide anion production was obtained with the highest BAC concentrations and similarly increased in a concentration dependent manner (Figure 3). On Chang cells, the highest ratio was obtained with BAC 10^−2^% (3.57, p<0.001 versus control) followed by BAC 5.10^−3^% (2.28, p<0.001), but results were not different from the control at the three other concentrations (ratios of 1.07, 1.01, and 1.04 for BAC 10^−3^%, 10^−4^%, and 10^−5^% [p=NS], respectively). On IOBA-NHC cells, increased ratios were obtained with BAC 10^−2^% (3.82, p<0.001) and BAC 5.10^−3^% (1.59, p<0.01). BAC 10^−3^% was not different from the control (0.93, p=NS), and BAC 10^−4^% and 10^−5^% significantly decreased superoxide anion production (ratios of 0.92 and 0.74 [p<0.05], respectively).

After 24 h of cell recovery (Figure 3), superoxide anion production increased with the five BAC concentrations on Chang cells. On IOBA-NHC cells, superoxide anion production was increased with BAC 5.10^−3^%, and decreased with the other four BAC concentrations.

### Apoptosis

#### Yopro-1 test

**Figure 4:**
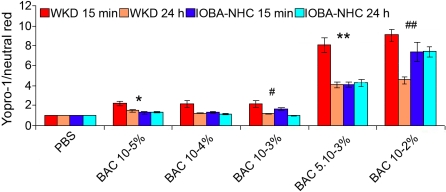
Apoptosis assay using the Yopro-1 test on Chang (WKD) and IOBA-NHC cells. This figure shows the results of the Yopro-1 apoptosis assay (microplate cytofluorometry) on Chang (WKD) and IOBA-NHC cells after two incubation times, 15 min of treatment with different BAC concentrations (WKD 15 min, IOBA-NHC 15 min) and 15 min of treatment with different BAC concentrations followed by 24 h of cell recovery in complete medium (WKD 24 h, IOBA-NHC 24 h). Note that the Yopro-1/neutral red ratio that is correlated to cell apoptosis consistently increased with BAC concentration on both cell lines. The asterisk symbols denote statistically significant differences between the two cell lines after 15 min of treatment (**p<0.001, *p<0.01), and the sharps (hash mark) denote statistically significant differences between the two cell lines after 24 h of cell recovery(##p<0.001, #p<0.05). BAC benzalkonium chloride; PBS, phosphate buffered saline. Means ± SEM.

After 15 min of cell exposure to the different concentrations of BAC, we observed that the Yopro-1/neutral red ratio consistently increased with BAC concentration (Figure 4).

On Chang cells, ratios were 9.04, 8, 2.17, 2.18, and 2.19 (p<0.001 versus control for all values) for BAC 10^−2^%, 5.10^−3^%, 10^−3^%, 10^−4^%, and 10^−5^%, respectively. On IOBA-NHC cells, lower ratios were obtained, 7.29 (p<0.001), 4.05 (p<0.001), 1.64 (p<0.001), 1.32 (p<0.05), and 1.29 (p=NS) for BAC 10^−2^%, 5.10^−3^%, 10^−3^%, 10^−4^%, and 10^−5^%, respectively.

After 24 h of cell recovery (Figure 4), the ratios decreased with the five BAC concentrations on Chang cells whereas only minor changes were observed on IOBA-NHC cells.

#### Annexin/V-7AAD staining

**Figure 5:**
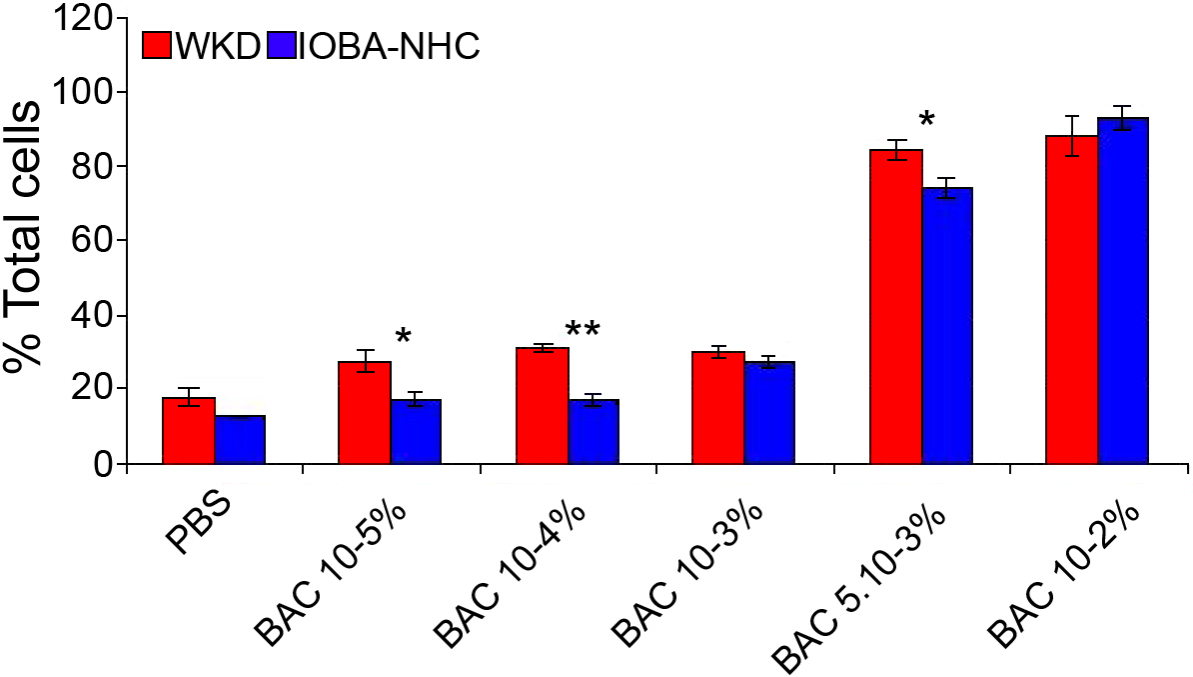
Total toxic effect evaluation using the Annexin/V-7AAD staining on Chang (WKD) and IOBA-NHC cells. This figure shows the total toxic effect (apoptotic and necrotic cells) using the Annexin/V-7AAD test (flow cytometry) after 15 min of treatment with different concentrations of BAC on Chang (WKD) and IOBA-NHC cells. Note that the same toxicity profiles were obtained on both cell lines with BAC concentration-dependent cell toxicity. The asterisk symbols denote statistically significant differences between the two cell lines (**p<0.001, *p<0.05). BAC, benzalkonium chloride; PBS, phosphate buffered saline. Means ± SEM.

For the total toxic effect evaluation (apoptotic and necrotic cells), results were expressed in percentage of cells engaged in a cell death process. With this global parameter, the same toxicity profiles were obtained on the two cell lines with BAC concentration-dependent cell toxicity (Figure 5). On Chang cells, the percentage of apoptotic or necrotic cells were 88.2% (p<0.001 versus control), 84.7% (p<0.001), 29.9% (p<0.01), 31.4% (p<0.01), and 27.3% (p<0.05) for BAC 10^−2^%, 5.10^−3^%, 10^−3^%, 10^−4^%, and 10^−5^%, respectively. Similar results were obtained on IOBA-NHC cells, with percentages of 92.9% (p<0.001), 74.1% (p<0.001), 27.5% (p<0.001), 17.1% (p=NS), and 17.4% (p=NS) for BAC 10^−2^%, 5.10^−3^%, 10^−3^%, 10^−4^%, and 10^−5^%, respectively.

**Figure 6:**
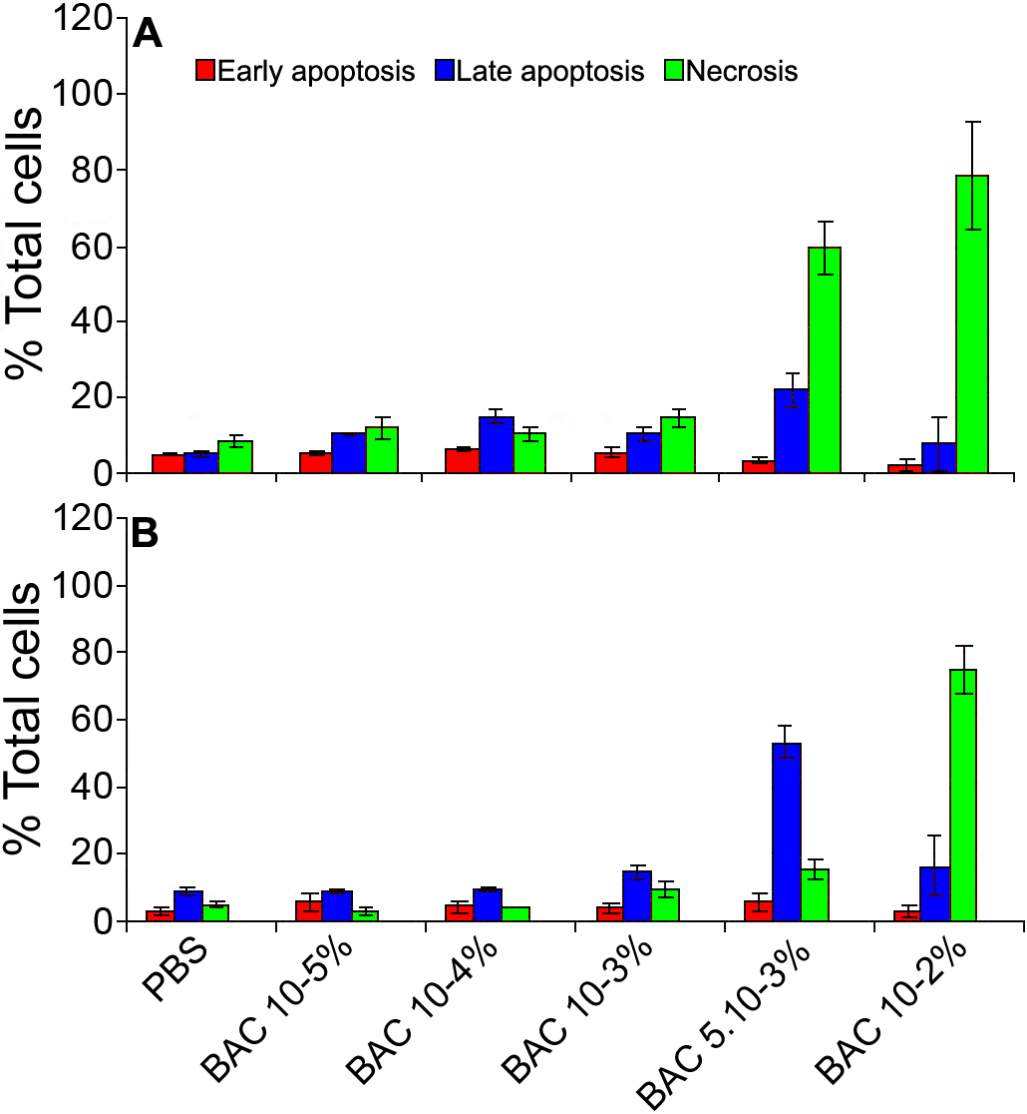
Discrimination of the early apoptotic, late apoptotic, and necrotic cell populations using the Annexin/V-7 AAD test on Chang (WKD) and IOBA-NHC cells. Annexin/V-7 AAD staining results (flow cytometry) show the early apoptotic, late apoptotic, and necrotic cell populations after 15 min of treatment with different concentrations of BAC on (**A**) Chang (WKD) and (**B**) IOBA-NHC cells. Note that BAC toxicity (late apoptosis) was found with BAC at 10^−5^% and 10^−4^% in Chang cells whereas on IOBA-NHC cells, the toxicity was only found with BAC at 10^−3^% and higher, and that with BAC at 5.10^−3^%, most of IOBA-NHC cells were in a late apoptotic process whereas most of Chang cells were necrotic. Statistical results are not shown (developed in the results section). BAC, benzalkonium chloride; PBS, phosphate buffered saline. Means ± SEM.

With the Annexin/V-7 AAD test, we also analyzed the early apoptotic, late apoptotic, and necrotic cell populations (Figure 6), discriminated as described previously. Particularly, we showed that on Chang cells, the late apoptotic cell population was significantly increased with BAC at 10^−5^% (10.4%, p<0.01 versus control) and with BAC at 10^−4^% (14.6%, p<0.001). Conversely, when concerning the early apoptotic and necrotic cell populations in Chang cells or the early apoptotic, late apoptotic, and necrotic cell populations in IOBA cells, no statistically significant difference was shown between these same BAC concentrations and PBS. With BAC 10^−3^%, the late apoptotic cell population was significantly increased in both Chang cells (10.3%, p=0.02 versus control) and IOBA-NHC cells (14.5%, p=0.042) whereas the early apoptotic and the necrotic cell populations were not significantly increased in both cell lines. In addition, no statistically significant difference was found between the two cell lines in the late apoptotic cell population with BAC 10^−3^%. With BAC 5.10^−3^%, on Chang cells, the percentages of late apoptotic cells and necrotic cells in Chang cells were 21.9% and 59.7% (p<0.05 versus control), respectively. On IOBA-NHC cells, there was a higher percentage of late apoptotic cells (53.1%, p<0.001 versus control) and a lower percentage of necrotic cells (15.4%, p<0.05 versus control) with BAC at 5.10^−3^%.

#### DNA content

**Figure 7:**
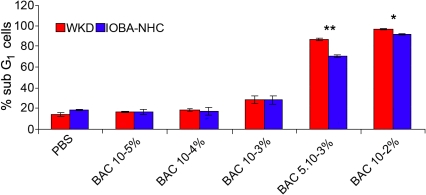
Alteration of DNA content analysis on Chang (WKD) and IOBA-NHC cells The alteration of DNA content was measured through the sub-G_1_ peak flow analysis (flow cytometry) on Chang (WKD) and IOBA-NHC cells after 15 min of cell exposure with the different concentrations of BAC followed by 24 h of cell recovery. Note that the same toxicity profiles were obtained on both cell lines with BAC concentration-dependent cell toxicity. The asterisk symbols denote statistically significant differences between the two cell lines (**p<0.001, *p<0.01). BAC, benzalkonium chloride; PBS, phosphate buffered saline. Means ± SEM.

Results were expressed in the percentage of sub-G_1_ events when compared to the total cell population after 15 min of treatment followed by 24 h cell recovery in normal culture medium (Figure 7). The same toxicity profiles were similarly obtained on both cell lines with this test. Indeed, percentages on Chang cells were 96.6% (p<0.001 compared with control), 86.8% (p<0.001), 28.8% (p<0.01), 17.9% (p=NS), and 16% (p=NS) for BAC 10^−2^%, 5.10^−3^%, 10^−3^%, 10^−4^%, and 10^−5^%, respectively, and on IOBA-NHC cells, the percentages were 91.3% (p<0.001%), 69.9% (p<0.001), 28.1% (p<0.05), 17.6% (p=NS), and 16.6% (p=NS), respectively, for the same BAC concentrations.

### Immunocytology

**Figure 8:**
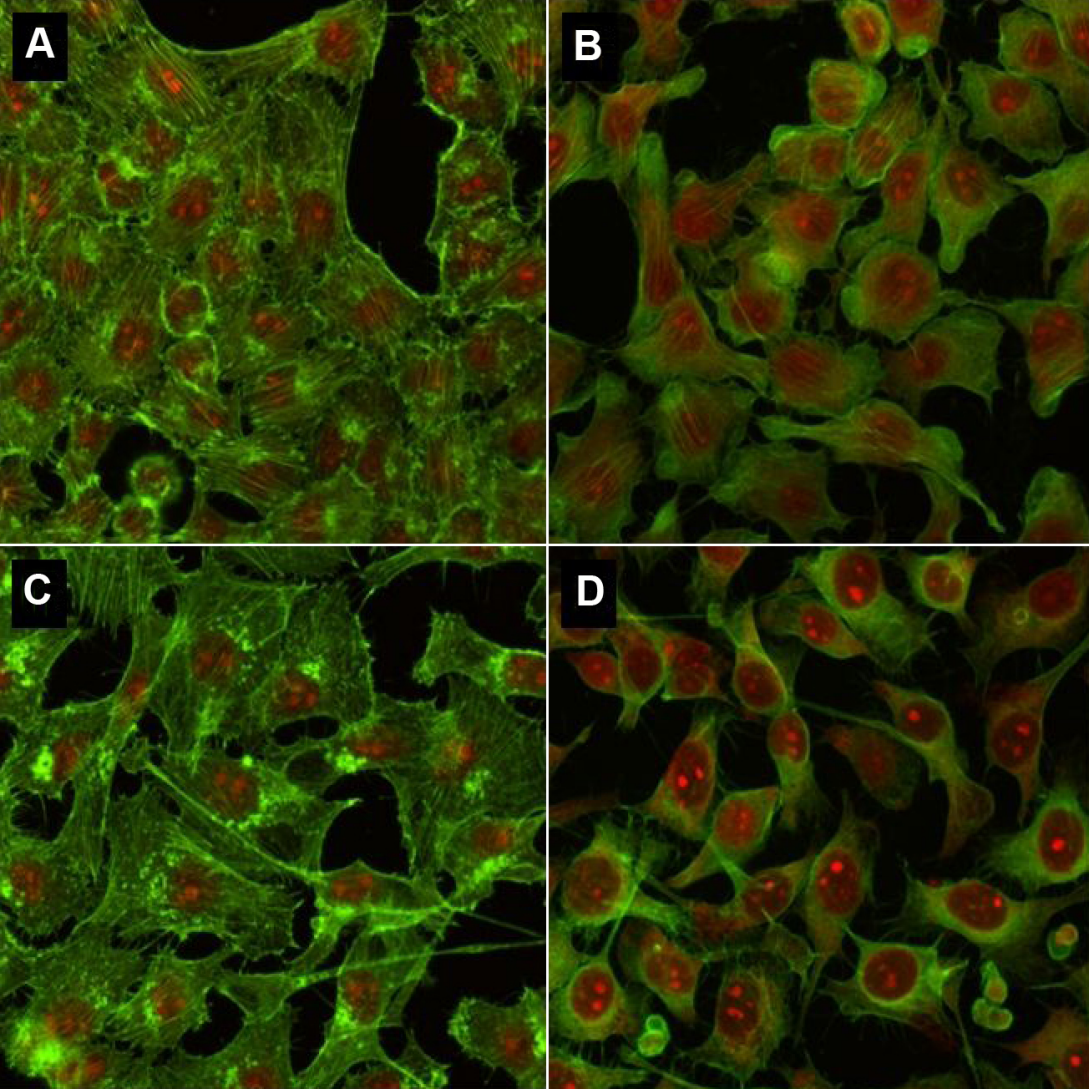
Standard immunofluorescence staining on Chang (WKD) and IOBA-NHC cells. Phalloidin and propidium iodide stainings show the morphology of Chang cells (**A**,**B**) and IOBA-NHC cells (**C**,**D**) after 15 min of treatment with solutions of PBS (**A**,**C**) and BAC 10^−2^% (**B**,**D**). Nuclei were stained in red by propidium iodide and cytoskeleton (F-actin) in green by phalloidin. Note the BAC-dependent increase of cell shrinkage on both cell lines. BAC, benzalkonium chloride; PBS, phosphate buffered saline.

Cell shrinkage increased in a BAC concentration-dependent manner after phalloidin and propidium iodide staining. Thus, we observed a high cell shrinkage with BAC 10^−2^% (Figure 8) and BAC 5.10^−3^% as well as intermediate changes with BAC 10^−3^% on both cell lines. No effect was shown with BAC 10^−4^% and 10^−5^%.

## Discussion

Cell cultures are generally considered to be a good alternative to animal studies for investigating the pathophysiology of human diseases. Particularly, cell lines permit easy, quick, cheap cell growth, small variations of experimental conditions, and better reproducibility than primary cultures. Currently, several continuous epithelial cell lines from the human conjunctiva have been developed. A few transformed cell lines have been reported and used for toxicological and functional studies [24-26], but only two untransfected cell lines have been developed, the Wong-Kilbourne derivative of Chang conjunctival cell line (clone 1 to 5c-4l American Type Culture Collection [ATCC, Manassas, VA]-certified cell line [CCL], 20.2) and the IOBA-NHC cell line. The Wong-Kilbourne derivative of Chang conjunctival cell line, the first continuous, untransfected epithelial cell line from normal human conjunctiva established, has been widely used in previous studies, particularly for the assessment of the toxicity of preservatives like benzalkonium chloride and preserved anti-glaucoma or anti-allergic eye drops [10-13,27,28]. Several functional studies have also been performed on this cell line [29,30], and its morphological and functional characteristics have been compared with primary culture of human conjunctival epithelium, underlining the immunomodulatory functions of both cell culture models in the localized inflammatory response [6,31]. However, the Wong-Kilbourne derivative of Chang conjunctival cells have HeLa marker chromosomes and the variant A of the enzyme, glucose-6-phosphate dehydrogenase, that could interfere with the interpretation of the results [7,32]. In 2003, Diebold et al. developed and characterized a spontaneously immortalized cell line from normal human conjunctiva called the IOBA-NHC cell line, which showed no other cell type contamination [8]. Like Chang cells, IOBA-NHC cells showed some morphological and functional characteristics of normal conjunctival epithelial cells like cytokeratin expression, presence of specialized adherent junctions between cells, and microvilli on the cell surface. However, like other cell lines, IOBA-NHC cells also showed some phenotypic and karyotypic differences with normal conjunctival epithelium. Nevertheless, this cell line has been used in functional and toxicological studies [33-38], but to our knowledge, no comparison of toxicological profiles of both Chang and IOBA-NHC cell lines have been performed. Thus, our purpose was to determine whether both cell lines are suitable and fully comparable for toxicological studies in vitro. Therefore, we compared both cell lines for the pro-necrotic, pro-apoptotic, and pro-oxidative effects of BAC, mainly used in ophthalmic solutions and which toxic effects are well known and have been widely reported on Chang cells [10,11].

In this study, all parameters of toxicity measured increased in a BAC dose-dependent manner on both cell lines. Indeed, the highest toxicity was obtained with the highest BAC concentrations on both Chang and IOBA-NHC cells after a 15 min exposition time and after 24 h of cell recovery. In addition, the BAC threshold concentration observed was BAC at 5.10^−3^% on both cell lines. These results were consistent with previous studies by De Saint Jean et al. [10] and Debbasch et al. [11] on Chang cells, who also noted a BAC dose-dependent increase in cell toxicity following exposure to BAC at different concentrations. In the present study, BAC toxicity was tested after two incubation times, 15 min of treatment and 15 min of treatment followed by 24 h of cell recovery in complete medium, to compare and understand both cell line behaviors and to approach the in vivo regeneration capacities of the conjunctival tissue. Thus, after the 24 h cell recovery period, we observed that BAC toxicity also increased in a concentration-dependent manner in the two cell lines and that the BAC threshold concentration was unchanged. With the neutral red and the H_2_DCF-DA tests, higher cellular damages were observed after the 24 h recovery period with BAC at 5.10^−3^% and above on both cell lines, their regeneration capacities were probably beyond their limits under these BAC concentrations. With the hydroethidine test that was used to detect the superoxide anion (O_2_^-^) production, cell toxicity increased after the 24 h recovery period on Chang cells but tended to decrease on IOBA-NHC cells except with a BAC concentration at 5.10^−3^%. Conversely, using the Yopro-1 test, we observed that the Yopro-1/neutral red ratio consistently decreased on Chang cells unlike on IOBA-NHC cells after 24 h of cell recovery. Thus, further experiments could be useful to study the cell characteristics potentially involved in these different cell behaviors such as the oxidative or anti-oxidative status of each cell line or the P_2_X_7_ cell death receptor expression after a single 15 min incubation time and after the 24 h recovery period.

In the present study, other small differences have been found between the two conjunctival cell lines. Indeed, BAC toxicity seemed to be higher on the Chang cell line than on the IOBA-NHC cell line, suggesting a higher sensitivity in Chang cells toward the toxic effects of BAC. With the sensitive Annexin/V-7AAD apoptosis test, a BAC toxicity (late apoptosis) was found with BAC at 10^−5^% and 10^−4^% in Chang cells whereas on IOBA-NHC cells, the toxicity was only shown with BAC at 10^−3^% and higher. With BAC at 5.10^−3^%, most of IOBA-NHC cells were in a late apoptotic process whereas most of Chang cells were necrotic, underlining the higher sensitivity of Chang cells. Several factors could explain thethese differences: the mitochondrial cells status, the expression or activation of cell death receptors (tumor necrosis factor, P2X7, or Fas receptors), or the factors or enzymes implicated in the oxidative stress (catalases, superoxide dismutases, glutathione peroxidases, glutathione) or in the apoptotic process (caspases, DNases). Another parameter that could explain these differences is the supplementation of IOBA-NHC cell culture medium with several components like mouse epidermal growth factor and hydrocortisone that could enhance the resistance of cells against BAC. In addition, with the neutral red test, cellular viability was significantly increased in IOBA-NHC cells with BAC at 10^−5^% and 10^−4^% after 15 min of cell treatment but mostly after 24 h of cell recovery. This effect could be explained by a higher cell growth of this cell line compared to Chang cells, but further experiments are required to confirm this hypothesis.

In the present study, the Wong-Kilbourne derivative of Chang conjunctival cells seemed to have a high sensitivity to BAC. These data suggest that the HeLa cell contamination to Wong-Kilbourne derivative of Chang conjunctival cells does not affect the usefulness of the cell line, supporting the use of Chang cells for toxicological, in vitro studies. Thus, as it has been previously reported, mild toxic effects of preservative or other potentially toxic agents can be accurately screened using this cell line [12,13,27,28,39] despite its contamination with HeLa cells. In addition, in this study, we showed a dose-dependent toxicity of BAC in IOBA-NHC cells, supporting the use of this cell line for toxicological in vitro studies as well, particularly for the comparison of apoptotic or oxidative effects of different ophthalmic medications. However, the interpretation of the studies performed on IOBA-NHC cells must consider the potential lower sensitivity of these cells and their culture medium composition. In every instance, toxicological or immunological studies require complementary tests and techniques to validate any in vitro experimental result, particularly if the aim is to confirm the innocuousness of an agent that could appear toxic on various models or using other techniques. Moreover, as Chang and IOBA-NHC cell lines are in vitro models, results obtained with each cell line cannot fully be extrapolated to in vivo conditions. In vivo, cells are protected with the action of the eyelids, the preocular mucin layer, and glycocalyx, and there is a permanent renewal of ocular surface epithelia. Tissues have high regeneration and defense capacities, and the conjunctival epithelium has a stratified structure that enhances protection of the ocular surface against preservative toxicity. Furthermore, our findings are insufficient to strictly validate the use of IOBA-NHC or Chang cells for toxicological studies. Validation of any new alternative method that has been defined by the demonstration of the reliability and relevance of a test method for a particular purpose must be demonstrated in an independent, scientifically sound validation program [40]. Currently, the only alternative method that has been validated as predictive of the ocular irritancy response is the rabbit Draize model [41], although this model is subjective and flawed. However, several alternative in vitro methods have been developed over the past 20 years to replace it [42].

In conclusion, this present study demonstrates that the comparison of BAC toxicity on both Chang and IOBA-NHC cell lines supported the use of IOBA-NHC cells for toxicological studies in vitro and validated previous studies performed on the Chang cells despite their reported contamination with HeLa cells. Thus, our findings confirm that both cell lines are useful for toxicological in vitro studies as their respective disadvantages are known and discussed. Particularly, IOBA-NHC cells, which showed no other cell type contamination, are cultured in a medium that is supplemented with epidermal growth factor (EGF), hydrocortisone, and cholera toxin, factors which may be potentially implicated in the cell resistance against a toxic agent like BAC. Further studies could be performed to determine whether the deprivation of each factor could modify cell functional behavior and morphologic characteristics. Other factors could influence cell sensitivity to xenobiotics like the oxidative or mitochondrial status of tested cells and the expression of death receptors that could be characterized in further studies. Moreover, comparison of the characteristics of both cell lines will deserve further interest to complete the validation of the usefulness of these cell lines for functional studies.
